# Rheumatism and Gout

**Published:** 1904-04-09

**Authors:** 


### RHEUMATISM AND GOUT.

(*Concluded from page* 11.)

**Gout.**—Luff[22] in discussing the diagnosis of joint diseases, mentions the arthritis due to septic absorption through the uterus or prostate, and mentions six severe cases which followed ovarectomy, whether produced by the operation or by the disease for which it was done is not clear. True gonorrhœal arthritis may follow infection by the gonococcus either from the urethra or the conjunctiva. In the treatment of gout he relies chiefly on colchicum, guiacum resin, and potassium citrate. The lithium salts do not restrain the conversion of the sodium biurate into crystalline form, and they frequently cause much cardiac depression. In spite of the bad effect of sodium salts in hastening the changes of the biurate, if there are no deposits the common soda gentian and nux vomica mixture taken before meals is most useful in stimulating the liver to its work. Similarly at the outset, a dose of blue pill, followed by Carlsbad salts, is to be recommended. In chronic gout, galvanism, preceded and followed by massage, often brings about a rapid reduction in size of the joints, and unless the disease is of very long-standing, radiant heat baths often start a steady improvement which goes on long after they cease to be given. Cataphoresis is also useful, the bicarbonate of potash being applied on a pad to the joint under the positive pole, with about 20 milleampéres of current. For prophylaxis 10 grains of guiacum resin may be given three times a day in a capsule. Toogood[23] notes the importance for diagnosis of the first appearance of the disease in the great toe, while the incidence of subsequent attacks is largely influenced by "occupation strains" in any joint. The flexion of the distal phalanx and the over-extension of the central one of the middle finger is also typical. Luff[24] speaks strongly on the injury done by mistaking rheumatoid arthritis for gout, and by giving a poor diet, which is ruinous in the former disease. He believes strongly in guiacol for rheumatoid arthritis, and says that if enough is given and for a sufficient period, it will generally arrest the disease. Indeed, the disease if taken early is curable. He gives the almost tasteless carbonate of guiacol in doses up to 20 grains three times daily in cachets for at least a year. [Other writers, however, find no difficulty in giving up to 20 or even 40 drops of guiacol, or pure creosote simply beaten up in milk three times daily for long periods to phthisical patients, and the smallest of these amounts is equal to 40 grains of the carbonate.] He also gives iodide with nux vomica and glycerophosphates. Douche-massage, radiant heat, "general" electric baths, and peat baths [or boxes filled with heated salt] are very helpful. The interest of investigators has been to some extent transferred from uric acid and the urates to the purin bodies, but A. C. Croftan[25] has carried out a series of experiments on the transformations of uric acid in the tissues, and he finds that (1) uric acid is normally destroyed in the human body by the aid of certain "ferments," the muscles destroying the greatest quantity, next the kidneys, then the liver, the spleen and finally the blood; (2) since the kidneys destroy it of themselves, the uric acid in the urine is not a measure of that in the blood, but failure of the kidney functions may lead to an excess of uric acid in the blood, *i.e.* renal disease may be a forerunner of gout; (3) the frequency of liver disorders before attacks of gout may imply the failure of the liver to destroy the circulating uric acid or that ingested in food; (4) the value of salicylates and dilute alkalies in gouty conditions is explained by the fact that they increase the liberation of oxygen from nucleo-proteids; (5) since the kidney can form oxalic acid out of uric acid, the presence of oxaluria in gout is also explicable. The experiments show, too, that uric acid is transformed into urea, and, therefore, uric acid is not a terminal product; (7) some of the organs at least which destroy uric acid also break up fats and sugars, hence when they are diseased we find together obesity, diabetes, and gout. Woods Hutchinson[26] points out that uric acid is not due to the imperfect change of proteids into urea, or from an excess of nitrogenous food, but rather from the nucleins alloxurs and purins in the food and tissues. It is clear that about half is due to the food and half to the destruction of nucleins in the tissue. The importance of uric acid, in spite of Haig and Garrod, is doubtful since it appears to be unirritating. Thus in leukæmia and in children it may be present in large amounts without any effects except for some mechanical disturbance when precipitated in the urine; and it has been injected into animals and given in their food with similar results. He regards the uric acid of gout like the phosphoric acid which accompanies it as the measure of the destruction of the leucocytes in response to an invasion of toxins. Most of these toxins are of intestinal origin. Hence the diet should be regulated to avoid fermentation and any excess of food beyond what the body can oxidise; and since it is only the endogenous half of the uric acid which is increased in gout, there is no reason for forbidding foods rich in purins and nucleins, such as sweetbreads. The function of the liver in gout, too, is merely negative, that is, it is unable to carry out its work as a poison filter and destroyer of toxins. Chalmers Watson,[27] too, argues that uric acid is not the primary factor in gout, and that it can be deposited in the tissues without any inflammatory reaction. Regarding the process in gout as due to intestinal toxins, he gives a minute account of the post-mortem findings in a case of acute gout in a fowl. Besides typical gouty changes in the tissues and synovia, he found features in the spleen and pancreas indicative of a bacterial invasion, and changes in the intestines and kidneys which pointed in the same direction. It was argued against him that the disease was really an acute nephritis, and that in birds the destruction of the kidneys leads to the accumulation of uric acid in the tissues, but Watson replies that the convoluted tubules were singularly healthy, and moreover uræmia does not seem to depend upon a retention of urea and similar bodies in the organism. The appearances in the tissues were certainly those of a general infectious process, and this is what he believes gout to be. Citarin has been praised as a remedy for gout. It is produced by the action of formaldehyde on citrate of soda, and the former is known to form a very soluble compound with uric acid. Liebholz[28] records most successful results, and advises its being given as early as possible in an acute attack as soon as the first symptoms appear, and in large doses, say 30 grains every six hours, which can be reduced to eight hours on subsequent days. The pain and swelling is brought to an end very rapidly, and no ill effects have been noticed except sometimes a slight diarrhœa.

[23] Clin. Jour., Oct. 7. [25] Med. Rec., Sept. 17. [24] Clin. Jour., Oct. 14. [25] Med. Rec. [26] Ibid., Oct. 10. [27] Brit. Med. Jour., Jan. 9. [28] Clin. Excerp., Nov.-Dec.

